# Clinicopathological features and prognostic factors for uterine myoma

**DOI:** 10.12669/pjms.38.6.5455

**Published:** 2022

**Authors:** Xiaoli Wu, Yanqing Zheng

**Affiliations:** 1 Xiaoli Wu, Department of Gynecology, Huzhou Central Hospital, Affiliated Hospital of Huzhou Normarl University, Huzhou 313000, Zhejiang Province, P.R. China; 2 Yanqing Zheng, Department of Gynecology, Huzhou Central Hospital, Affiliated Hospital of Huzhou Normarl University, Huzhou 313000, Zhejiang Province, P.R. China

**Keywords:** Uterine myoma, Enucleation of Uterine myoma, Total Hysterectomy, Pelvic Adhesion, Prognostic Risk Factors

## Abstract

**Objectives:**

To investigate the clinicopathological features of patients with uterine myoma and to analyze and summarize factors affecting patient prognosis.

**Methods:**

This study retrospectively investigated records of uterine myoma patients treated at Huzhou Central Hospital between June 2018 and May 2020. A total of 149 patients were included in this study, including 121 patients with good prognosis and 28 patients with poor prognosis. Clinical and pathological characteristics, including age, average body mass index (BMI), menopausal status, average lesion diameter, presence of hypertension or diabetes mellitus, operation method, myoma type, location, and quantity, number of fibroids, and presence of pelvic adhesion were analyzed via multivariate analysis.

**Results:**

Among the 149 patients with uterine uterine myoma, 92 had anterior wall uterine myoma, 36 had posterior wall uterine myoma, and 21 had uterine fundus uterine myoma. In terms of location, 94 cases were intramural and 55 cases were subserosal. Quantity-wise, 107 had 1-3 myomas while 42 had four or more. Moderate or more severe pelvic adhesions were present in 33 cases. Univariate analysis showed significant differences in age, operation method, myoma type, myoma location, myoma number, and pelvic adhesion severity between patients with good and poor prognosis. Multivariate analysis showed that age, surgical method, uterine myoma type, uterine myoma location, uterine myoma quantity, and pelvic adhesion severity were risk factors affecting the prognosis.

**Conclusion:**

Many prognostic factors, including age, operation method, myoma type, myoma location, myoma number, and pelvic adhesion severity are linked to uterine myoma patient prognosis.

## INTRODUCTION

Uterine myoma, also known as leiomyoma or fibroids, is the most common benign tumor of the female reproductive system. uterine myoma patients generally show no initial obvious signs, but aging can bring abnormal uterine bleeding, pelvic compression symptoms, pain, and impacted fertility. uterine myoma is the main underlying cause for clinical hysterectomy, which in turn has a significant adverse impact on women’s reproductive health, social and medical resource consumption, and the health economy at large. At present, the specific pathogenic mechanism of uterine fibroids is not well known but is believed to be related to a variety of factors, including diet, age, hypertension, diabetes, sex hormone levels, and familial genetics. uterine myoma is mostly treated with surgery,^1^ such as open uterine myoma enucleation, laparoscopic uterine myoma enucleation, open total hysterectomy, and laparoscopic total hysterectomy. However, these different surgical methods have different therapeutic effects and different prognoses.^2^,^3^ As such, this study reviewed the clinicopathological features of uterine myoma patients and analyzed factors potentially impacting patient prognosis.

## METHODS

This study retrospectively investigated clinical records of uterine myoma patients treated at Huzhou Central Hospital between June 2018 and May 2020. A total of 149 patients were included, featuring 121 patients with good prognosis and 28 patients with poor prognosis.

### Inclusion criteria:

• Presence of a complete clinical history and obvious clinical manifestations.
• uterine myoma diagnosis

### Exclusion criteria:

• Abnormal blood coagulation.
• Presence of severe infectious diseases and malignancies.
• Presence of severe abnormalities in cardiopulmonary function or liver and kidney function.

This study was approved by the medical ethics committee of Huzhou Central Hospital (Approval number: L210517, Date: 2021 May 28).

### Data on the following variables were collected:

age, BMI, menopausal status, lesion average diameter, presence of hypertension or diabetes mellitus, operation method, myoma type and location, presence and number of uterine fibroids, and pelvic adhesion information. Data was analyzed using SPSS 22.0 statistical software. Counting data were expressed as [n (%)] and compared using χ^2^ tests, while factors affecting patient prognosis were analyzed by multivariate logistic regression. P values less than 0.05 were considered statistically significant.

## RESULTS

Patient age ranged from 32 to 75 years, with an average age of 60.83±8.76 years. The mean BMI was 23.9±1.38 kg/m^2^. Average lesion diameter was 42.13±4.11 mm. Of the 149 total patients, 29 underwent open myomectomy, 51 underwent laparoscopic myomectomy, 23 underwent open hysterectomy, and 46 underwent laparoscopic hysterectomy. Anterior wall myoma was found in 92 cases, posterior wall myoma in 36 cases, and fundus myoma in 21 cases. Regarding myoma location, 94 cases were intramural and 55 cases were subserosal. Myoma count was three or less in 107 cases and four or more in 42 cases. Mild or less severe pelvic adhesions were found in 116 cases and moderate or more severe pelvic adhesions were found in 33 cases. Of all the patients, 121 had good prognosis and 28 had poor prognosis.

No significant difference in average BMI, average lesion diameter, or hypertension and diabetes mellitus incidences was noted between patients with good and poor prognosis. However, significant differences were found for age, operation method, myoma type, myoma location, myoma count, and pelvic adhesion severity between the two groups (Table-I). Age, operation method, myoma type, myoma location, myoma number, and pelvic adhesion severity were found to be risk factors affecting uterine myoma prognosis (Table-II).

**Table I:** Univariate analysis of related factors affecting the prognosis of uterine myoma (n).

Factor		n	Good prognosis group (n=121)	Poor prognosis group (n=28)	χ²	P
Age	≥60 years	72	47	25	23.169	<0.001
	<60 years	77	74	3		
BMI	≥24 kg/m²	95	74	19	0.251	0.617
	<24 kg/m²	54	45	9		
Menopausal status	Yes	124	101	23	0.029	0.865
	No	25	20	5		
Diameter of lesion	≥40mm	114	91	23	0.609	0.435
	<40mm	35	30	5		
Hypertensive	Yes	24	18	6	0.722	0.395
	No	125	103	22		
Diabetes mellitus	Yes	16	14	2	0.465	0.495
	No	133	107	26		
Operation method	Open myomectomy	29	17	12	40.169	<0.001
	Laparoscopic enucleation of uterine myoma	51	49	2		
	Open hysterectomy	23	11	12		
	Laparoscopic total hysterectomy	46	44	2		
Myoma type	Anterior wall	92	82	10	36.715	<0.001
	Posterior wall	36	32	4		
	Fundus uteri	21	7	14	
Myoma location	Intramural	94	69	25	10.162	0.001
	Subserosal	55	52	3		
Number of myomas	1-3	107	100	7	37.326	<0.001
	≥4	42	21	21		
Pelvic adhesion	None to mild	116	108	8	48.564	<0.001
	Moderate to severe	33	13	20		

**Table II:** Multivariate analysis of related factors affecting the prognosis of uterine myoma.

Factor	B	S.E.	Wald	df	P	OR	95%CI
Age	-2.1	0.762	7.588	1	0.006	0.122	0.027~0.546
Operation mode	-0.636	0.297	4.589	1	0.032	0.53	0.296~0.947
Myoma type	1.069	0.394	7.37	1	0.007	2.911	1.346~6.296
Myoma location	2.217	0.768	8.336	1	0.004	9.179	2.038~41.342
Number of myomas	-1.79	0.63	8.085	1	0.004	0.167	0.049~0.573
Pelvic adhesion	-2.313	0.685	11.405	1	0.001	0.099	0.026~0.379

## DISCUSSION

Morphologically, uterine myomas are mainly composed of connective tissue and smooth muscle. Since it can involve parts of the endometrium, uterine myoma may also lead to pathological changes, such as endometrial hyperplasia and endometrial cancer.^4^ Pharmacological interventions are restricted to growth inhibition rather than tumor removal, and surgery is still a preferred method of uterine myoma treatment.^5^,^6^ Hysterectomy is considered a traditional surgical intervention for uterine myoma, but the severity of this option has driven researchers to search for alternative options.^7^,^8^ More recently, myomectomy has become prioritized, with the overall goal of preserving uterine integrity. However, although myomectomy has achieved some clinical efficacy, patient prognosis following this procedure still varies.^9^,^10^ This shift in clinical intervention policy has also highlighted the importance of early diagnosis using a combination of clinical manifestation information and imaging examinations.^11^,^12^

This study showed that age equal to or greater than 60 was a risk factor affecting uterine myoma prognosis. This effect is potentially due to an age-related decline in physical and immune functions that results in a greater risk of endometrial hyperplasia caused by stimulation, as well as in greater physiological impact from surgical intervention. Our results are supported by previous studies. A study of 110 patients with uterine myoma also showed that age was positively correlated with tumor volume,^13^ while a study of 875 patients with endometrial hyperplasia and 263 patients with endometrial adenocarcinoma concluded that patient age is an important prognostic factor for endometrial cancer that is independent of other parameters.^14^

This study also found that uterine myoma patient prognosis is worse after open surgery as compared to laparoscopic surgery. Laparoscopic surgery methods are generally considered to be more accurate, leading to less adhesion, less local bleeding, and more thorough flushing of the pelvic and abdominal cavities. A clinical meta-analysis showed that laparoscopic surgery had lower incidence of complications and shorter postoperative hospital stay than laparotomy, furthermore, this analysis found that prognosis for cases involving the posterior wall and fundus was worse than cases involving the anterior wall.^15^ This is possibly due to the fact that the bladder of the forearm myoma generally needs to be filled to an appropriate filling degree, while the tissues and organs around anterior wall myomas are mainly physiological uterine and bladder tissues located far away from intestine and sacrum, therefore lowering risk of complications.^16^

This investigation shows that pelvic adhesion is a prognostic factor of uterine myoma, which is consistent with previous studies. Previous study, investigating primary endometrioid carcinoma with and without myometrial infiltration, as well as 20 cases of regional lymph node metastasis found that myometrial infiltration was an independent prognostic parameter of endometrioid carcinoma associated with pelvic and/or paraaortic lymph node metastasis risk.^17^ Finally, four or more myomas in a patient were found to be a risk factor, probably due to a fact that in cases of multiple myomas, intervention requires more incisions on the surface of the uterus, leading to higher risk of post-operative adhesions.^18^

### Limitations of the study

It must be noted that this study may deviate from other research findings due to a small, ethnically and regionally homogenous patient pool. Multicenter, large sample size, and longer follow-up are needed to better assess patient prognosis.

## CONCLUSION

This study analyzed the pathological characteristics of patients with uterine myoma, which may help improve the accuracy of disease diagnosis. Age, operation method, myoma type, myoma location, myoma numbers, and pelvic adhesion are risk factors affecting the prognosis of uterine myoma, and can provide a reference for clinical treatment.

### Authors’ contributions:

**XW:** Conceived and designed the study.

**XW & YZ:** Collected the data and performed the analysis.

**XW:** Was involved in the writing of the manuscript and is responsible for the integrity of the study.

All authors have read and approved the final manuscript.
